# Procaine at Low Concentration Modulates Mitochondrial Features in Cultured Fibroblasts and Myoblasts: A Clue to the Cellular Mechanisms Underlying Neural Therapy

**DOI:** 10.3390/ijms27209327

**Published:** 2026-10-20

**Authors:** Chiara Rita Inguscio, Giada Remoli, Ilaria Scambi, Franco Donati, Manuela Malatesta

**Affiliations:** 1 Department of Neurosciences, Biomedicine and Movement Sciences, University of Verona, I-37134 Verona, Italy; chiararita.inguscio@univr.it (C.R.I.); giada.remoli@univr.it (G.R.); ilaria.scambi@univr.it (I.S.); 2 Studio Donati Franco, I-40128 Bologna, Italy; dottdonatifranco@gmail.com

**Keywords:** neural therapy, local anesthetic, low procaine concentration, mitochondrial shape, mitochondrial cristae, mitochondrial membrane potential, fibroblasts, myoblasts, transmission electron microscopy

## Abstract

Procaine is a local anesthetic used at low concentrations in a complementary medical approach, neural therapy, due to its ability to promote antioxidant and anti-inflammatory response. Previous biochemical studies showed that low concentrations of procaine may also stimulate mitochondrial function. In the present study, low procaine concentrations were used to investigate in vitro the effects on mitochondria of human fibroblasts and mouse myoblasts. Histochemical, ultrastructural, and biomolecular techniques were employed. After 24 h from procaine treatment, mitochondrial membrane potential was unaltered in both cell types, but some mitochondrial features showed cell-specific modifications. In detail, in fibroblasts, procaine induced decreased mitochondrial cristae length and cellular adenosine triphosphate (ATP) levels. In myoblasts, procaine induced modifications of mitochondrial shape and oxidative phosphorylation subunit amount, together with increased cellular ATP levels. Most modifications were restored at 48 h. To explain the differential response of the two cell lines, we hypothesize a species-specific sensitivity to procaine or a cell-specific sensitivity related to the different extent of the endoplasmic reticulum, which is especially susceptible to procaine. Taken together, the results of this pilot study show that procaine at low concentration exerts a modulatory effect on mitochondrial features, opening the way to further studies aimed at elucidating the cellular pathways involved in the beneficial effects observed in procaine-based neural therapy.

## 1. Introduction

Procaine, first synthesized in 1905, belongs to a class of analgesic medications known as amino ester local anesthetics, and consists of a lipophilic aromatic ring, a terminal amine, and an intermediate ester or amide group [1].

The fundamental mechanism of procaine analgesia is the blockade of neurotransmission via inhibition of sodium channels along nerve fibers: procaine reversibly binds to active sites of neuronal voltage-gated sodium channels inhibiting their open configuration, thus blocking sodium influx and suppressing neurotransmission [2]. In addition, procaine inhibits potassium (K^+^) conductance in cell membrane channels, thus acting as a depolarizing agent [3], and inhibits adenosine triphosphate (ATP)-dependent calcium (Ca^2+^) uptake, thus modulating intracellular calcium homeostasis [4]. Procaine interacts hydrophobically and electrostatically with membrane phospholipids and cholesterol, modifying the physicochemical properties of the lipid bilayer and inhibiting membrane functions [5]. Moreover, procaine modifies the molecular packing of membrane phospholipids, thus altering membrane fluidity [6,7].

Despite its analgesic properties, procaine has clinical limitations such as slow onset, short length of activity and systemic toxicity. In fact, once in the organism, procaine is rapidly degraded into para-aminobenzoic acid and diethylaminoethanol, mainly due to the action of plasma esterases [8,9,10]. Procaine can also cause adverse effects, from hypersensitivity (with anaphylactoid-like symptomatology) to the compromission of multiple organ systems (called local anesthetic systemic toxicity), due to the binding of procaine to beta-adrenergic receptors in addition to voltage-gated sodium channels [1]. This mediates cardiotoxic (e.g., arrhythmia, hemodynamic instability, heart failure and cardiac arrest) and neurotoxic (e.g., tinnitus, blurred vision, tremors, seizures, unconsciousness and respiratory arrest) effects [1]. Thus, procaine has shortly been replaced by other local anesthetics and, nowadays, it is administered mostly to induce numbness in few specific medical procedures (e.g., dental extraction).

However, from the mid-twentieth century, procaine at very low concentrations (generally 0.5–1% or even lower) has been increasingly used in neural therapy, also referred to as therapeutic local anesthesia [11]. This is a complementary medical approach aimed at resolving chronic pain and restoring organism homeostasis by targeting the autonomic nervous system (reviews in [11,12,13]).

The first evidence of the therapeutic effects of procaine administered at low concentration dates to 1906, when its anti-inflammatory properties were demonstrated [14]. Subsequently, several authors reported beneficial effects in different pathological conditions such as trigeminal neuralgia, migraine, fractures, dystrophic disorders, allergy, alopecia, hypertension, nephrolithiasis, Raynaud syndrome, and cancer [11,13,14,15,16]. Neural therapy was found to be especially effective in treating neurological, cardiovascular, locomotor, dermatological, and gastrointestinal disorders in the elderly [16].

It has been demonstrated that, at low concentrations, procaine may influence different biological functions in various cell types, promoting antioxidant effects [17,18,19,20], modulating cytokine metabolism with anti-inflammatory response [21,22,23,24,25], inducing DNA demethylation [26,27,28,29,30], downregulating the mitogen-activated protein kinase signaling pathways (in particular the extracellular signal-regulated kinase, ERK and p38) [31], inactivating the Janus kinase 2/signal transducer and activator of transcription 3 pathway [32], and reducing cholesterol synthesis and esterification [33,34,35]. These properties led some authors to consider procaine as a “geroprotective” agent, although this definition remains highly controversial [36,37]. Nonetheless, scientific research demonstrated that the multiple biological actions of procaine at low concentrations may possibly account for the beneficial clinical effects observed in a variety of pathological conditions [11,13].

Mitochondria play a pivotal role in maintaining cell homeostasis; in fact, although universally known as the cellular powerhouses producing ATP through respiratory activity, they are also involved in multiple signaling pathways. They contribute to the regulation of redox balance [38]; function as biosynthetic centers catabolizing nutrients, generating precursors for macromolecules, compartmentalizing metabolites to preserve redox homeostasis, and managing metabolic waste [39]; and play a primary role in calcium buffering regulating the local Ca^2+^ concentration in cellular compartments [40]. Mitochondria are involved in regulation of gene expression through the synthesis of specific metabolites (e.g., acetyl-CoA, alpha-ketoglutarate) and by epigenetic control, in the frame of a bidirectional crosstalk [41,42,43]. Mitochondria regulate cell division, differentiation and migration not only by producing ATP, but also through their dynamic shape changes (fusion and fission) and the modulation of calcium and reactive oxygen species [44,45]. They are also involved in multiple regulated cell death forms, such as apoptosis, pyroptosis and ferroptosis [46,47]. Under non-lethal apoptotic stress, mitochondria trigger signaling affecting genome stability, senescence, immunity, and inflammation [48]. Recent evidence demonstrated that mitochondria or mitochondrial components may be transferred among cells through various routes (tunneling nanotubes, extracellular vesicles, gap junctions, mitochondrial extrusion), thus improving energy status, redox and metabolic homeostasis, and immune defense [49].

Mitochondria are very sensitive to stressing agents, which may induce dysfunction and damage of these organelles, even leading to pathological conditions such as cardiovascular, neurodegenerative and metabolic diseases, and cancer [50]. However, stressing conditions may also stimulate mitochondria to elicit cytoprotective signaling [51,52].

Importantly, mitochondria were found to be affected by procaine. At the high concentrations used for anesthesia, procaine acts as an uncoupling agent perturbing calcium homeostasis. In detail, procaine acts on Ca^2+^ channels [53], thus causing an overload of calcium in the endoplasmic reticulum [54] and mitochondria [55]: this Ca^2+^ accumulation may result in mitochondrial dysfunction and loss of membrane potential until the whole organelle is damaged [55,56]. However, biochemical studies performed in the eighties of the last century demonstrated that procaine, when administered at low concentrations, not only maintains mitochondrial function but may even stimulate oxidative phosphorylation (in mitochondria extracts from liver and brain [57,58]), electron transport activity (in mitochondria extracts form liver and heart [59]), and pyruvate dehydrogenase activity (in mitochondria from isolated adipocytes [60]).

The purpose of the present study is to deepen the knowledge on the effects of procaine at a low concentration on mitochondria. To this end, histochemical, ultrastructural, and biomolecular techniques were used in a multidisciplinary approach to provide information on fine structural and functional features of mitochondria in fibroblasts and myoblasts treated in vitro with low procaine concentrations. This knowledge could shed light on the possible role of these organelles in the observed beneficial effects of procaine-based neural therapy.

## 2. Results

### 2.1. Cell Viability Assays

The 3-(4,5-dimethylthiazol-2-yl)-2,5-diphenyltetrazolium bromide (MTT) assay was used to evaluate the cell viability of human fibroblasts and mouse myoblasts following treatment with different procaine concentrations in order to identify the maximum concentration that did not cause a significant increase in cell death in both cell types after 24 and 48 h.

**Figure 1:**
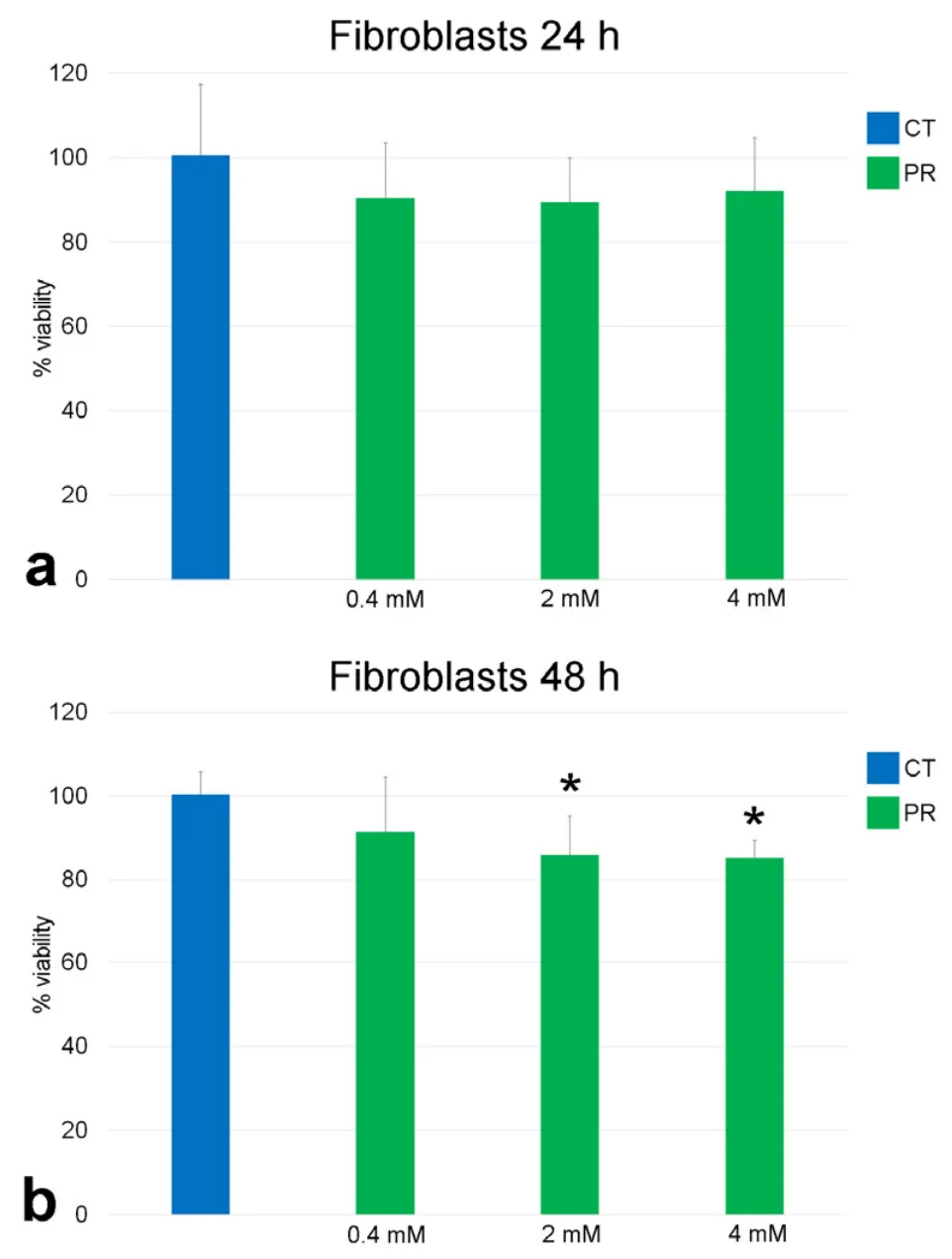
Mean ± SE values of cell viability as assessed by the MTT assay on fibroblasts at 24 h (**a**) and 48 h (**b**) after administration of different procaine concentrations. Data were obtained from three independent experiments (n = 3). Asterisks indicate significant difference in comparison with the control (CT): 2 mM procaine (PR)-treated samples vs. CT, *p* = 0.04; 4 mM PR-treated samples vs. CT, *p* = 0.01.

**Figure 2:**
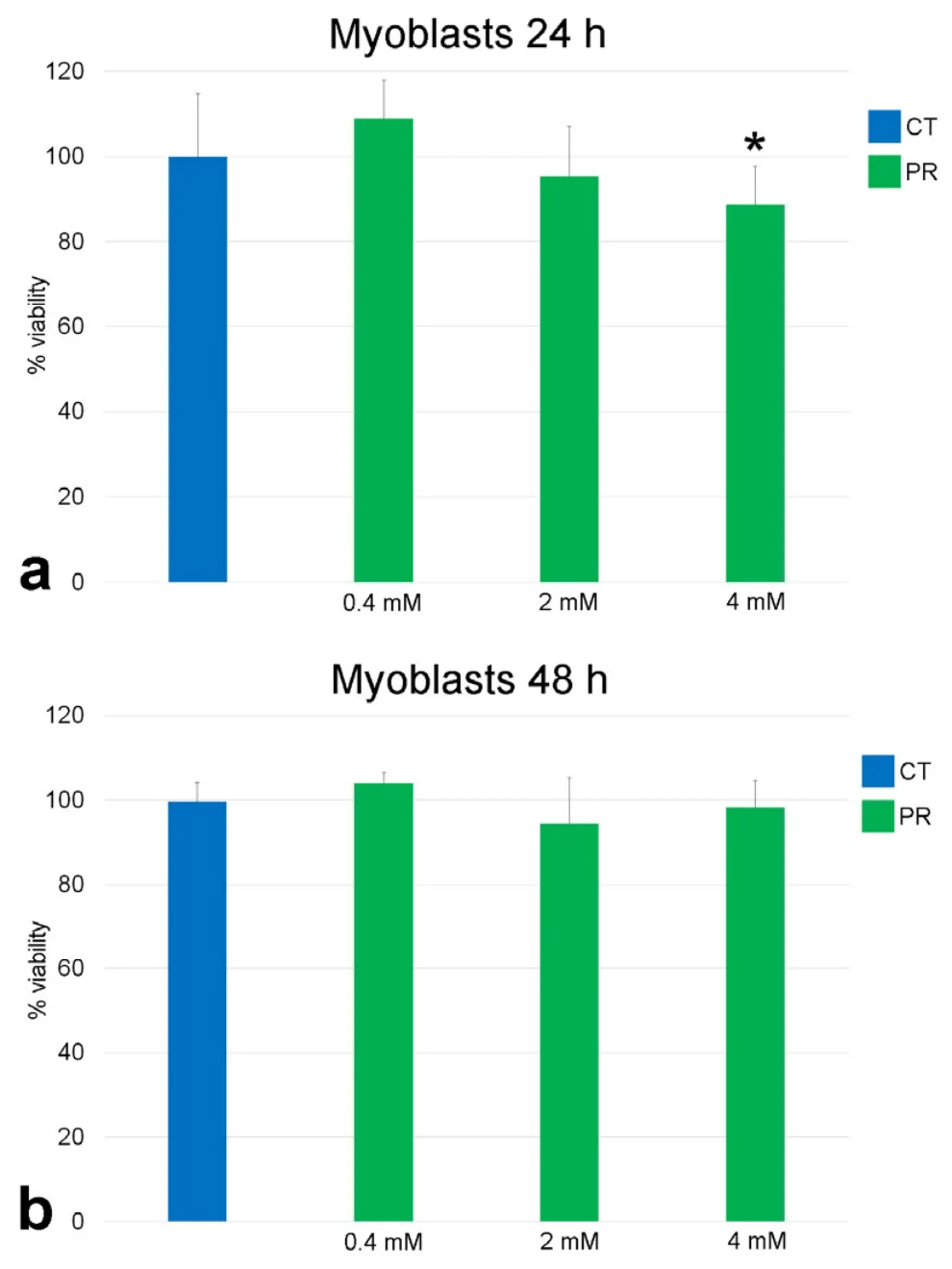
Mean ± SE values of cell viability as assessed by the MTT assay on myoblasts at 24 h (**a**) and 48 h (**b**) after administration of different procaine concentrations. Data were obtained from three independent experiments (n = 3). The asterisk indicates significant difference in comparison with the control (CT): 4 mM procaine (PR)-treated samples vs. CT, *p* = 0.04.

Figure 1 and Figure 2 report mean ± standard error of the mean (SE) values of the MTT assay in fibroblasts and myoblasts, respectively. Statistical analysis revealed that the safe procaine concentration for both cell lines was 0.4 mM (corresponding to 0.01%).

Since the MTT assay primarily measures cellular reducing/metabolic capacity and is highly influenced by mitochondrial and cytosolic redox metabolism, the safety of 0.4 mM procaine was further assessed by the trypan blue exclusion test, which is independent of mitochondrial metabolism and is based on the dye capability to cross the plasma membrane of dead cells. The counting of trypan blue-positive (dead) cells showed values below 2% in all samples at both 24 h (mean ± SE values: 1.43 ± 0.32% in control and 1.35 ± 0.67% in procaine-treated fibroblasts; 1.90 ± 0.43% in control and 1.15 ± 0.31% in procaine-treated myoblasts) and 48 h (0.50 ± 0.22% in control and 0.40 ± 0.30% in procaine-treated fibroblasts; 1.09 ± 0.29% in control and 0.50 ± 0.16% in procaine-treated myoblasts). No statistically significant difference was found between control and treated fibroblasts or myoblasts at both 24 h (*p* = 0.75 and *p* = 0.31, respectively) and 48 h (*p* = 0.81 and *p* = 0.21, respectively).

Based on these results, the experiments were carried out using 0.4 mM procaine.

### 2.2. S-Phase Evaluation

**Figure 3:**
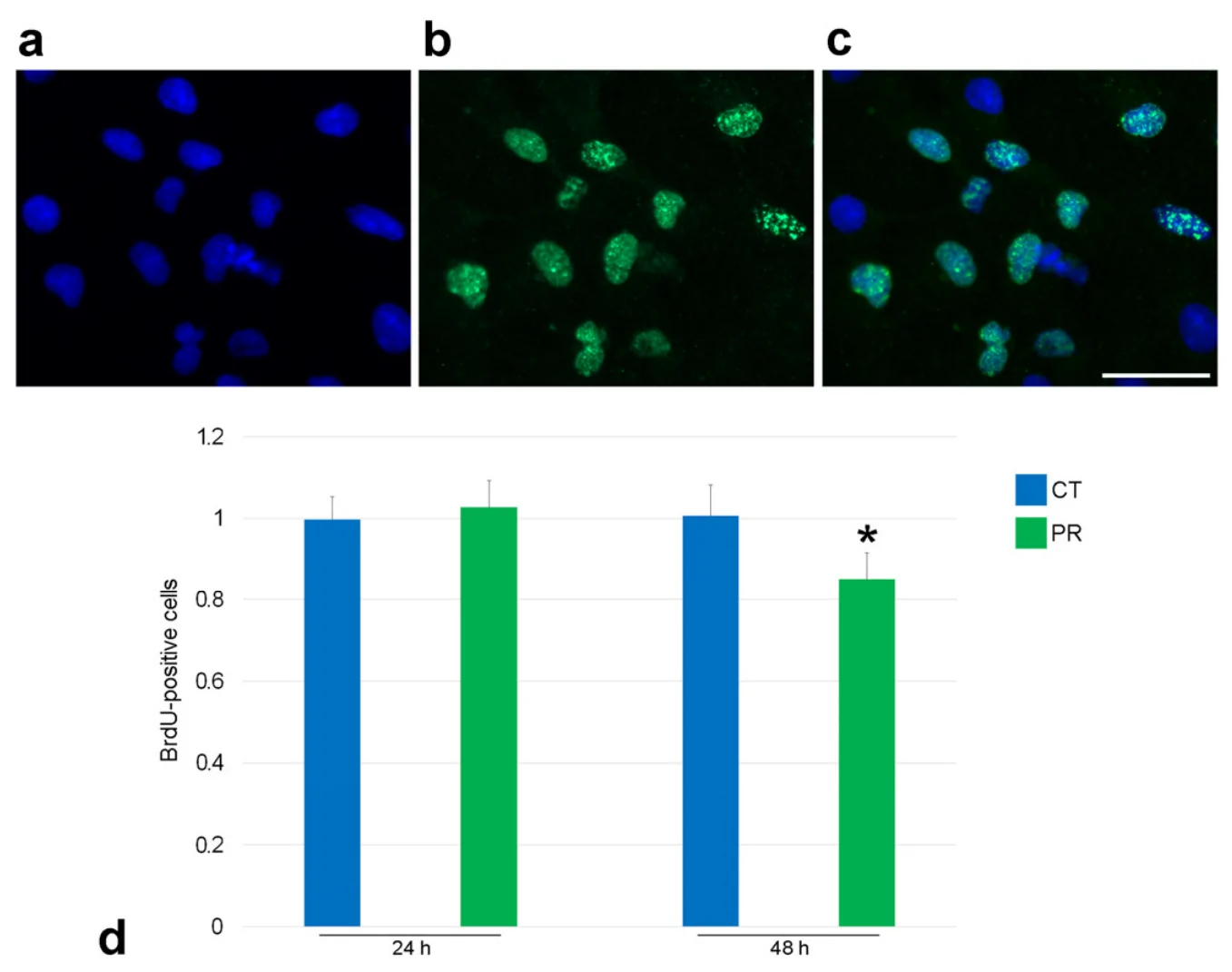
Representative images of fibroblasts immunolabelled for BrdU (**a**), counterstained for DNA with Hoechst 33342 (**b**); merged image in (**c**). Bar: 50 μm. (**d**) Mean values ± standard error of the mean of BrdU-positive cells 24 h and 48 h after the treatment. Values are expressed as fold change with respect to the control value. Data were obtained from three independent experiments (n = 3). The asterisk indicates significant difference from the respective control (CT): procaine (PR)-treated samples vs. CT, *p* = 0.01.

**Figure 4:**
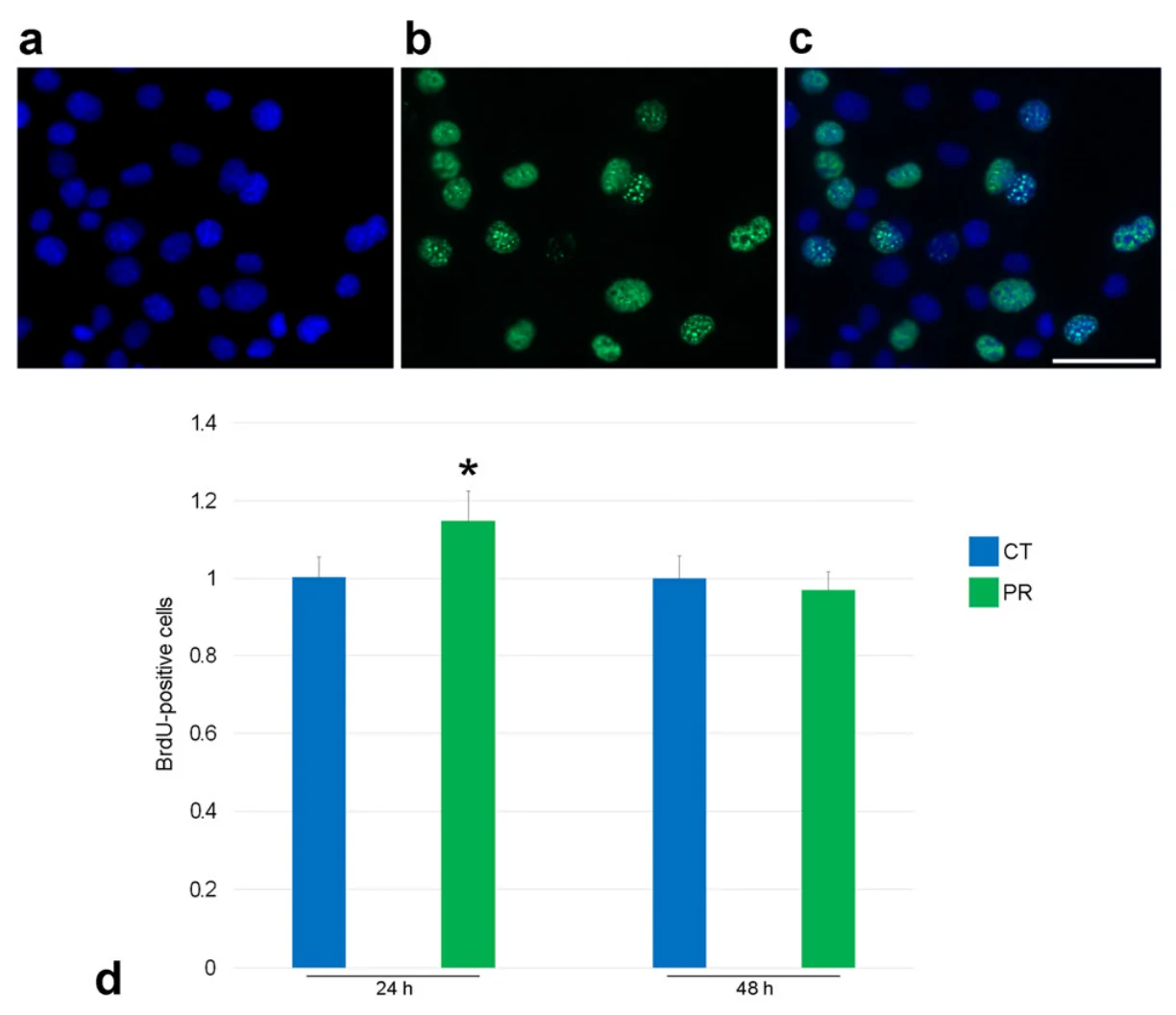
Representative images of myoblasts immunolabelled for BrdU (**a**), counterstained for DNA with Hoechst 33342 (**b**); merged image in (**c**). Bar: 50 μm. (**d**) Mean values ± standard error of the mean of BrdU-positive cells 24 h and 48 h after the treatment. Values are expressed as fold change with respect to the control value. Asterisk indicates significant difference from the respective control. Data were obtained from three independent experiments (n = 3). The asterisk indicates significant difference from the respective control (CT): procaine (PR)-treated samples vs. CT, *p* < 0.001.

Proliferating cells were detected and quantified by using 5-bromo-2′-deoxyuridine (BrdU), a thymidine analog that is incorporated into newly synthesized DNA during the S-phase. After specific immunolabelling, BrdU-positive cell nuclei were green-fluorescing, while nuclear DNA was blue-fluorescing after counterstaining with Hoechst 33342 (Figure 3a–c and Figure 4a–c). In fibroblasts, the percentage of BrdU-positive cells did not change (*p* > 0.05) after 24 h from the administration of 0.4 mM procaine, whereas it significantly decreased in treated cells after 48 h (*p* = 0.01) (Figure 3d). In myoblasts, the BrdU-positive cell rate significantly increased after 24 h from procaine administration (*p* < 0.001) and was similar to the control after 48 h (Figure 4d).

### 2.3. Mitochondrial Membrane Potential Assay

**Figure 5:**
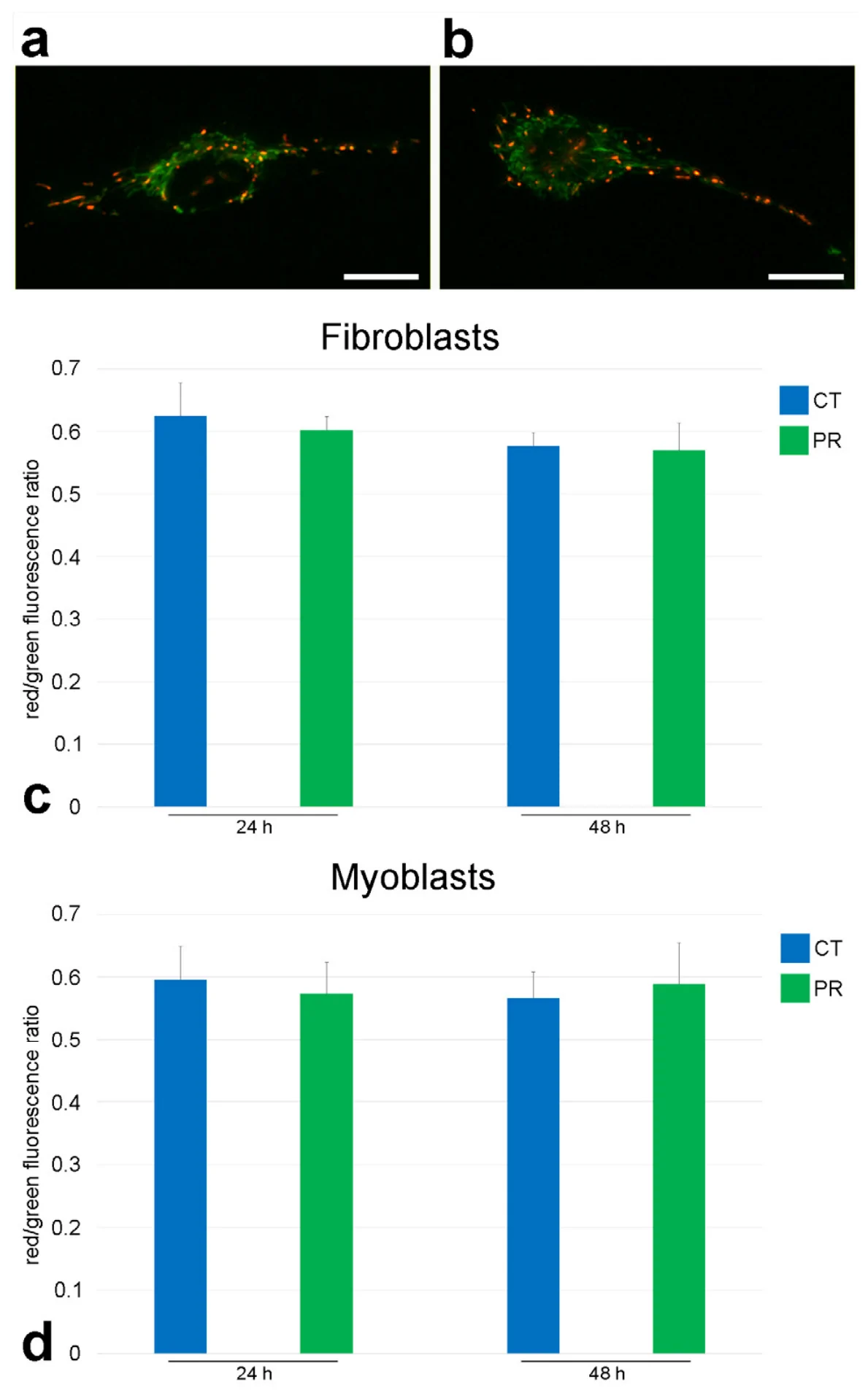
Representative images of a fibroblast (**a**) and a myoblast (**b**) stained with JC-1. Bars: 20 μm. (**c**,**d**) Mean values ± standard error of the mean of green/red fluorescence ratio at 24 h and 48 h after procaine treatment. No significant difference was found. Data were obtained from three independent experiments (n = 3). CT, control; PR, procaine-treated samples.

Mitochondrial membrane potential was monitored using the dye JC-1, which accumulates in mitochondria where it emits either red or green fluorescence depending on the membrane potential: the red signal indicates polarized (highly functional) mitochondria, while the green signal the depolarized (less functional) ones (Figure 5a,b). The green-to-red fluorescence ratio was digitally evaluated (Figure 5c,d). No significant difference (*p* > 0.05) in mitochondrial membrane potential was found between treated and control fibroblasts or myoblasts at 24 h and 48 h from procaine administration.

### 2.4. Transmission Electron Microscopy (TEM) Analysis

**Figure 6:**
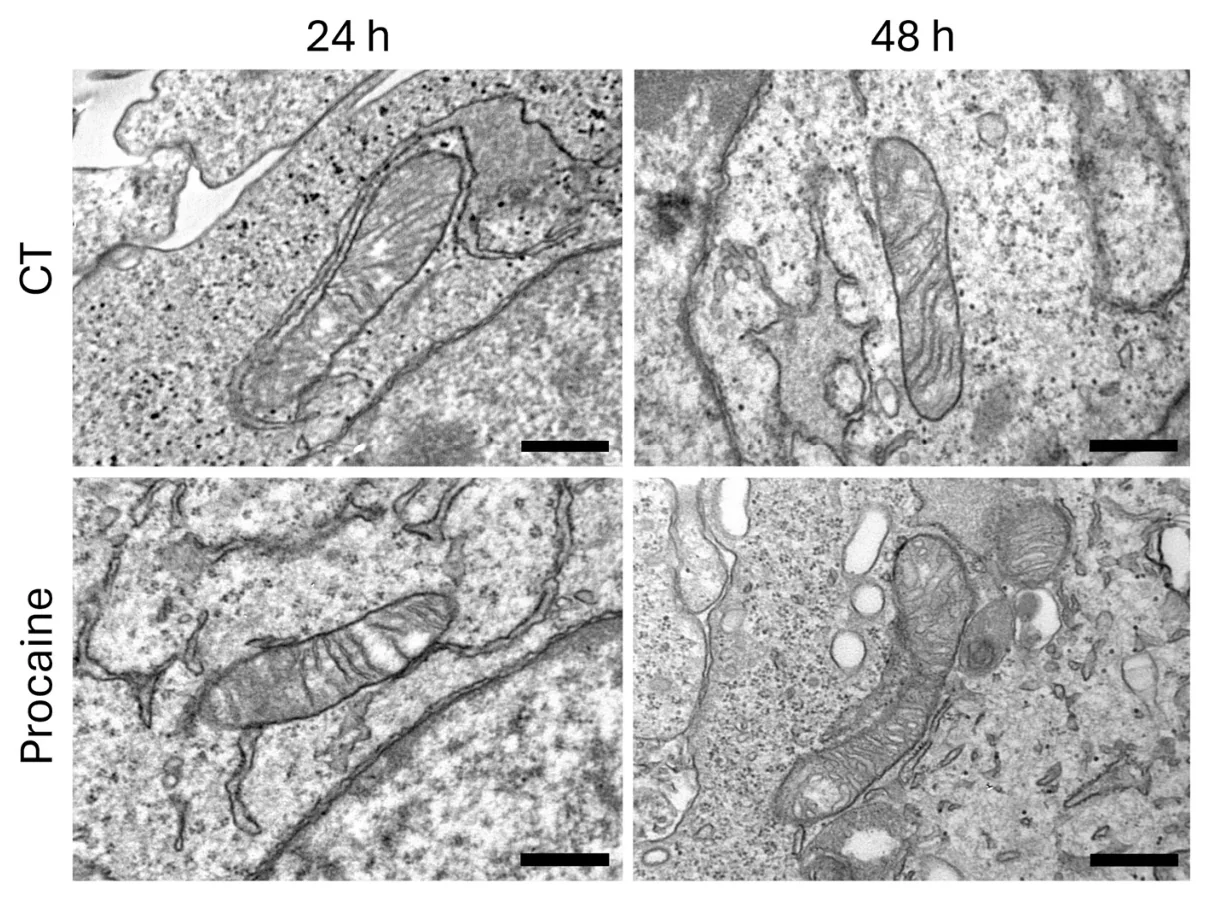
Transmission electron micrographs of mitochondria of control (CT) and procaine-treated fibroblasts at 24 h and 48 h. Bars: 400 nm.

**Figure 7:**
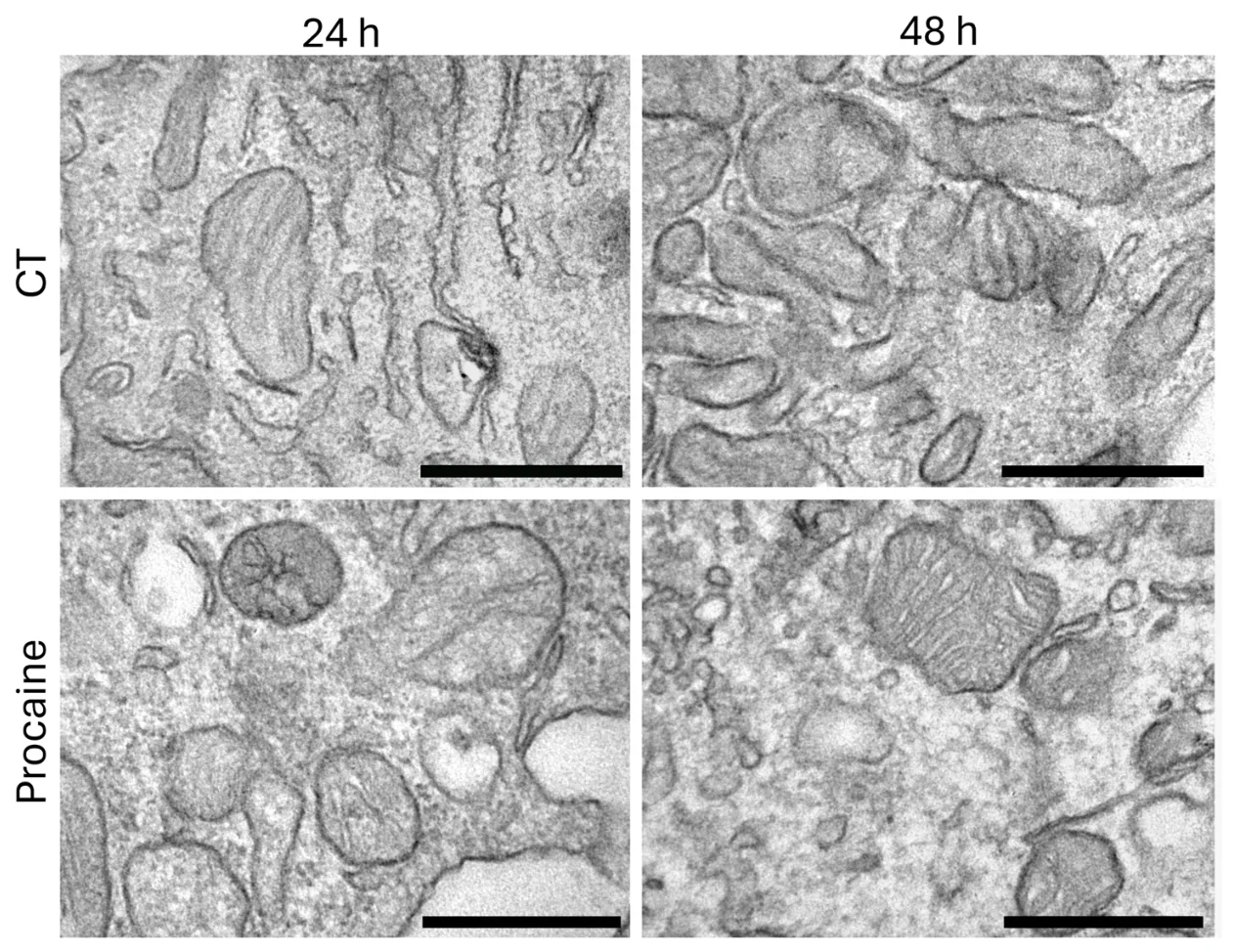
Transmission electron micrographs of mitochondria of control (CT) and procaine-treated myoblasts at 24 h and 48 h. Bars: 400 nm.

TEM observations revealed well-preserved elongated mitochondria rich in lamellar cristae (Figure 6 and Figure 7) in all cell samples. No morphological alteration was observed in fibroblast or myoblast mitochondria after 24 h or 48 h from procaine administration.

**Figure 8:**
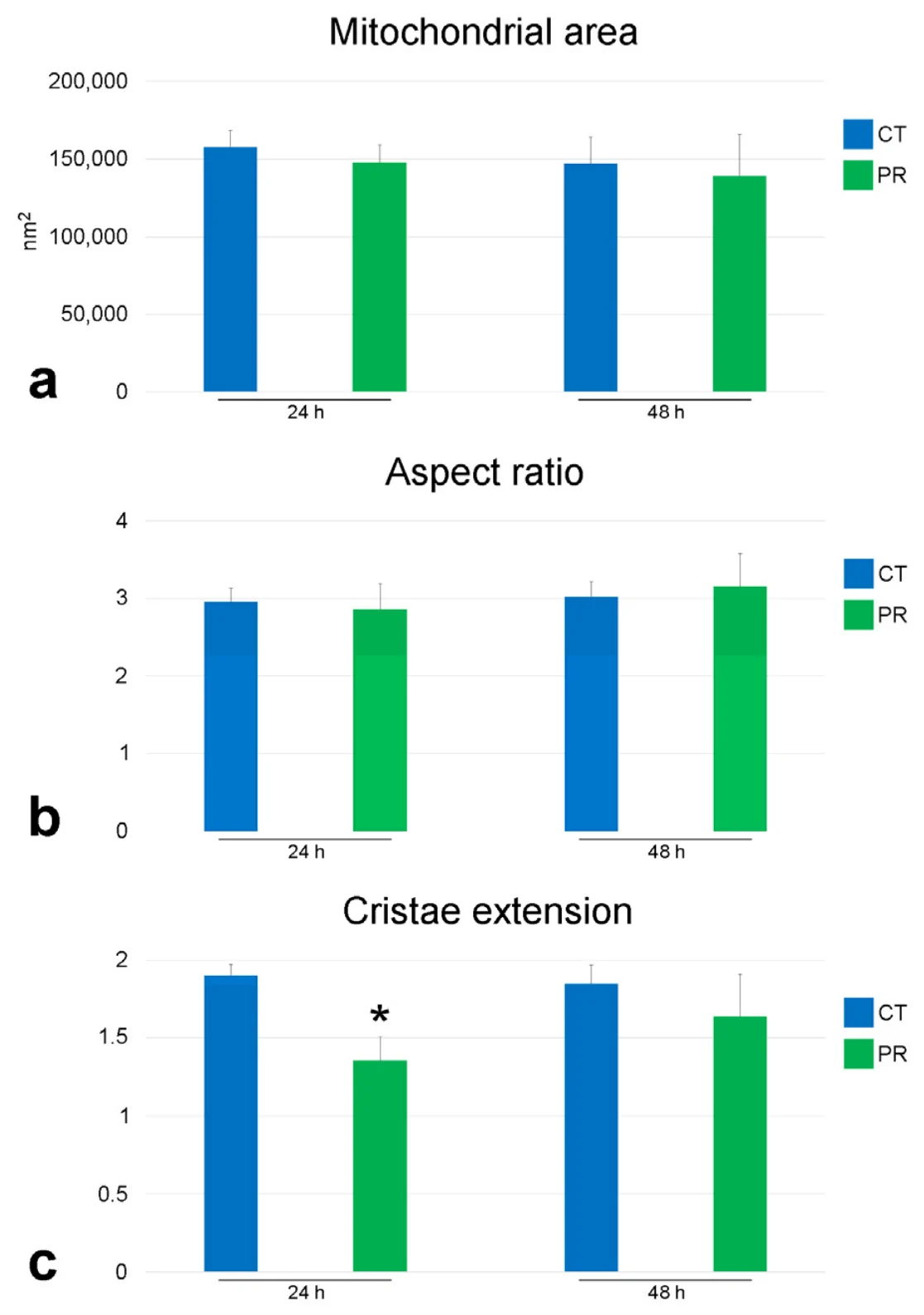
Mean values ± standard error of the mean of mitochondrial area (**a**), aspect ratio (**b**), and cristae extension (**c**) measured in fibroblasts 24 h and 48 h after procaine treatment. Data were obtained from three independent experiments (n = 3). The asterisk indicates significant difference from the respective control (CT): procaine (PR)-treated samples vs. CT, *p* = 0.04.

**Figure 9:**
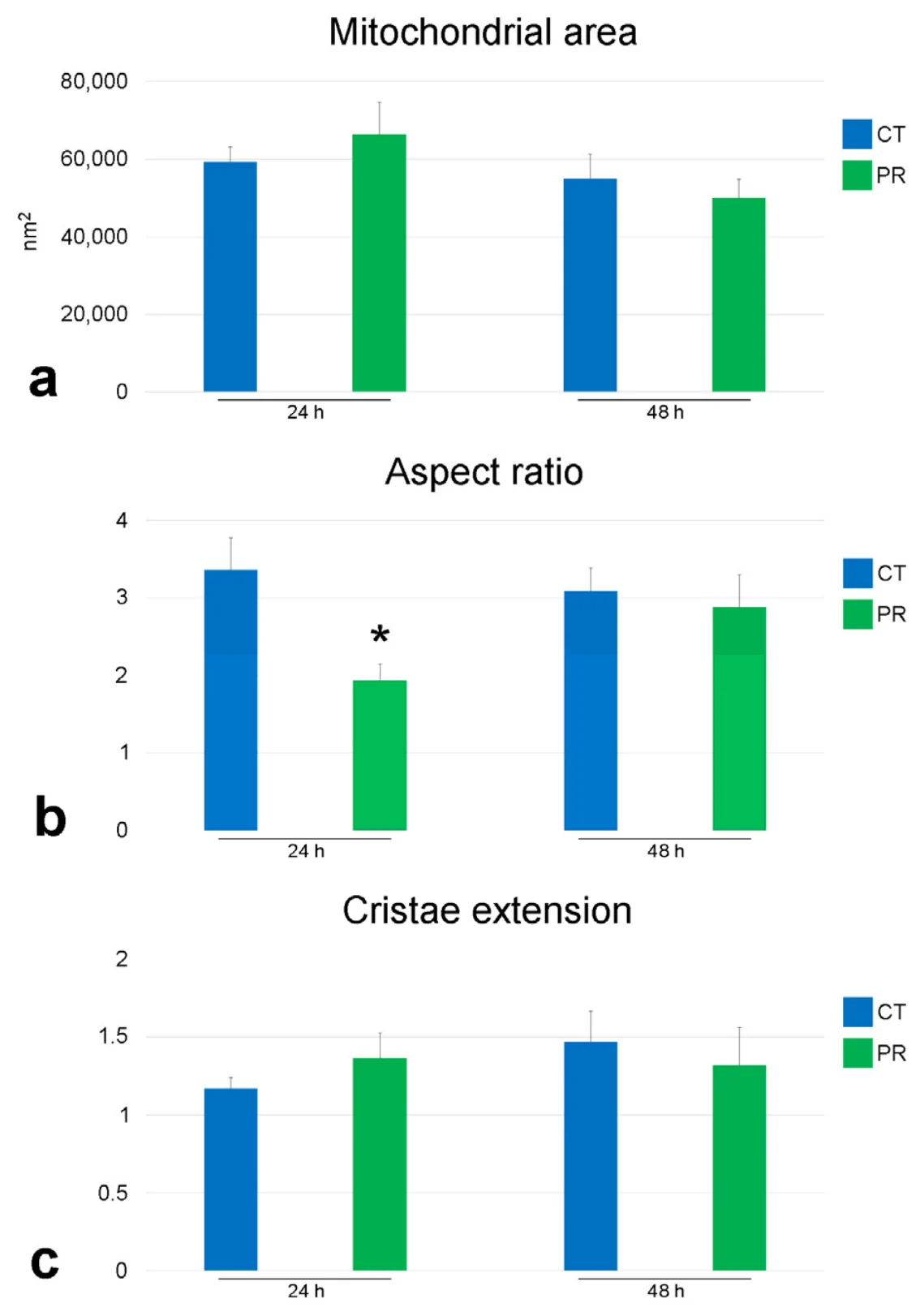
Mean values ± standard error of the mean of mitochondrial area (**a**), aspect ratio (**b**), and cristae extension (**c**) measured in myoblasts 24 h and 48 h after procaine treatment. Data were obtained from three independent experiments (n = 3). The asterisk indicates significant difference from the respective control (CT): procaine (PR)-treated samples vs. CT, *p* = 0.01.

However, the morphometric analysis revealed some quantitative modifications. In particular, 24 h after procaine administration, fibroblasts showed a significant reduction in the length of the mitochondrial cristae (*p* = 0.04) (Figure 8), while in myoblasts the mitochondrial aspect ratio (that quantitatively measure mitochondrial elongation) significantly decreased (*p* = 0.01), indicating a more roundish shape of the organelle (Figure 9). All modifications disappeared after 48 h from procaine administration, when the values were similar to the respective controls (*p* > 0.05).

### 2.5. Cellular ATP Assessment

**Figure 10:**
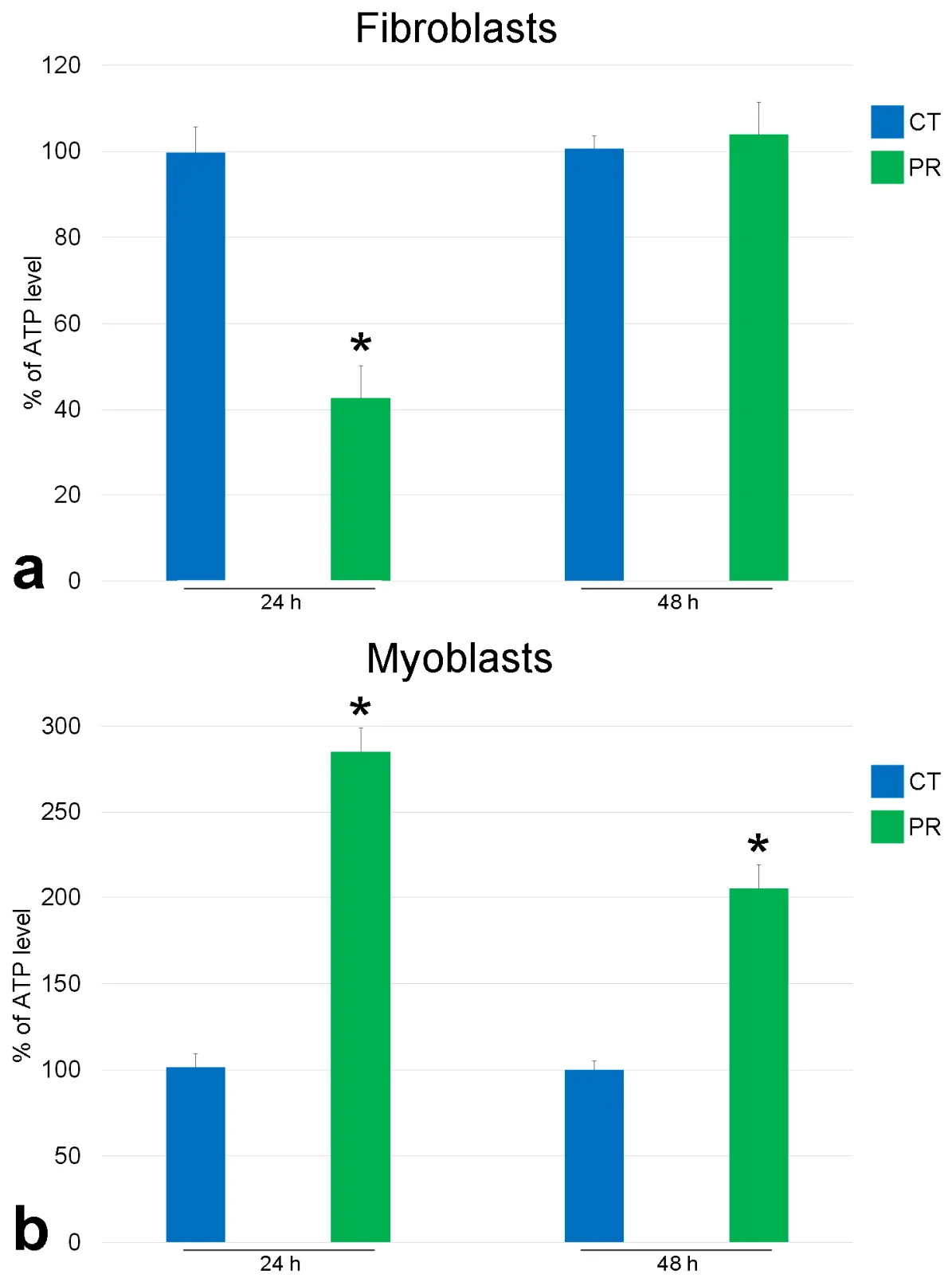
Mean ± SE values of cellular ATP levels in fibroblasts (**a**) and myoblasts (**b**) at 24 h and 48 h after procaine administration. Data were obtained from three independent experiments (n = 3). Asterisks indicate significant difference from the respective control (CT): fibroblasts 24 h, procaine (PR)-treated samples vs. CT, *p* = 0.01; myoblasts 24 h, procaine (PR)-treated samples vs. CT, *p* = 0.01; myoblasts 48 h, procaine (PR)-treated samples vs. CT, *p* = 0.01.

Cellular ATP was quantified by a bioluminescence assay at 24 h and 48 h after procaine administration as an index of ATP production and consumption. The percentage values were normalized to the control, which was fixed as 100%. Statistical analysis revealed modifications of treated cells in comparison to their respective controls (Figure 10). In detail, in fibroblasts a significant reduction in ATP was found after 24 h (*p* = 0.01), with a full restoration to the control values after 48 h (*p* > 0.05). In myoblasts, a significant increase in comparison to control was found at both 24 h (*p* = 0.01) and 48 h (*p* = 0.01) from procaine administration.

### 2.6. Quantification of the Mitochondrial Oxidative Phosphorylation (OXPHOS) Complex Subunits

**Figure 11:**
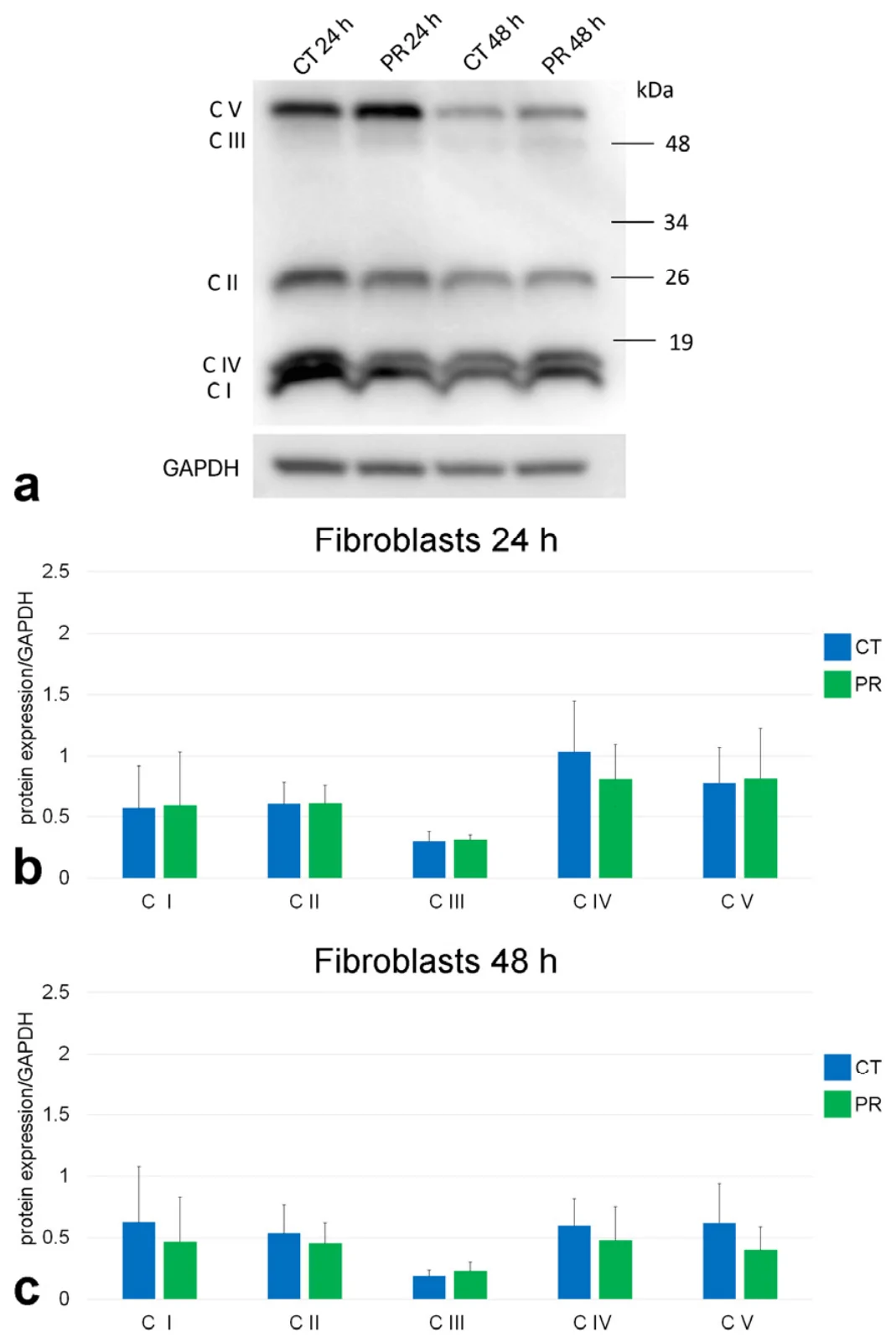
Quantification of the expression of OXPHOS complex subunits. (**a**) Representative immunoblot of CI, CII, CIII, CIV, and CV in control and treated fibroblasts 24 h and 48 h after procaine administration. (**b,c**) Mean values ± SE of CI, CII, CIII, CIV, and CV expression 24 h (**b**) and 48 h (**c**) after procaine administration. No statistically significant difference was found. Data were obtained from three independent experiments (n = 3). CT, control; PR, procaine-treated samples.

**Figure 12:**
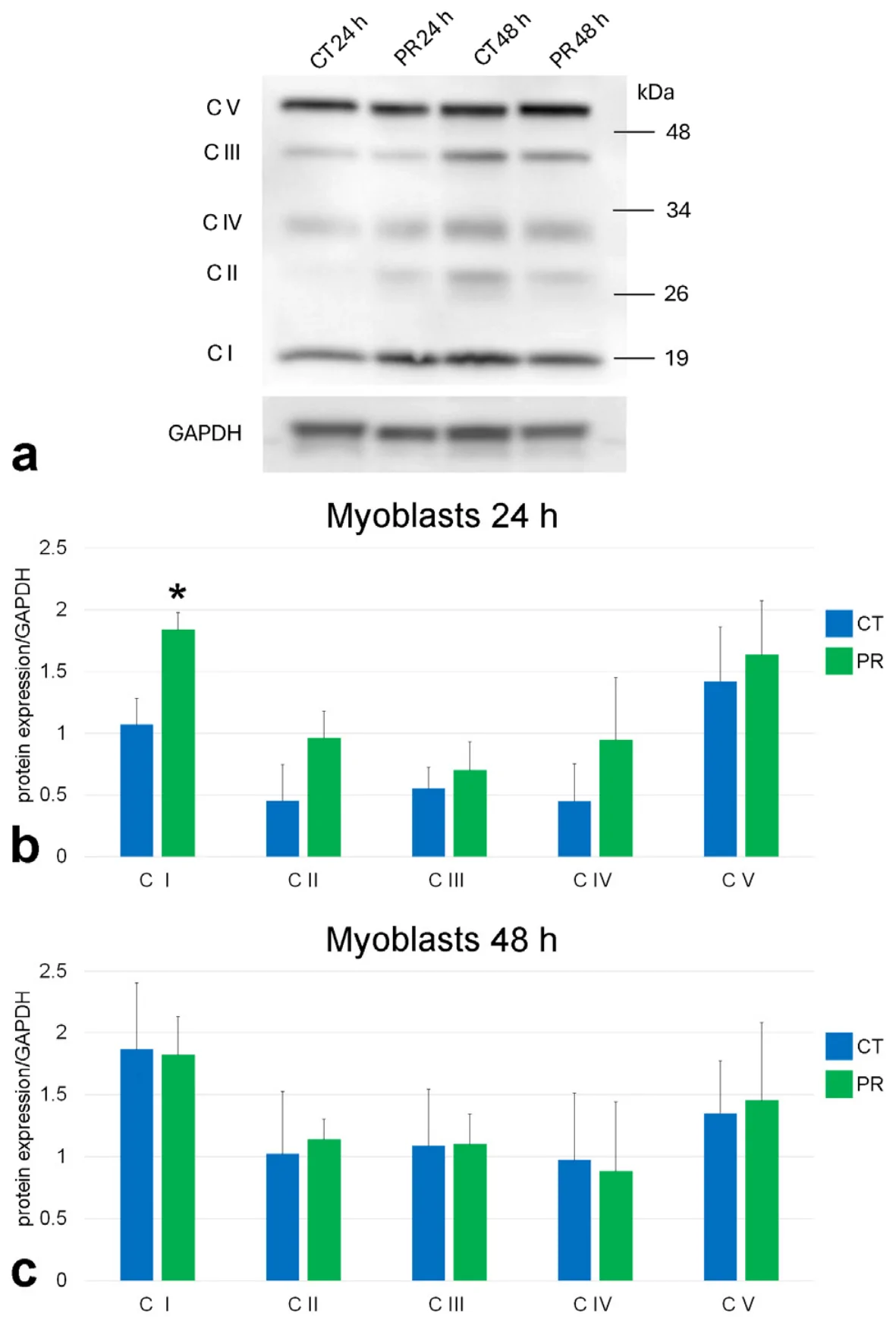
Quantification of the expression of OXPHOS complex subunits. (**a**) Representative immunoblot of CI, CII, CIII, CIV, and CV in control and treated myoblasts 24 h and 48 h after procaine administration. (**b,c**) Mean values ± SE of CI, CII, CIII, CIV, and CV expression 24 h (**b**) and 48 h (**c**) after procaine administration. Data were obtained from three independent experiments (n = 3). The asterisk indicates significant difference from the respective control (CT): procaine (PR)-treated samples vs. CT, *p* = 0.04.

The expression of the OXPHOS complex subunits (CI, CII, CIII, CIV, and CV) was measured by Western immunoblotting in fibroblasts and myoblasts after 24 h and 48 h from procaine administration. Statistical analysis revealed no significant difference for any OXPHOS complex subunits in fibroblasts at both 24 h and 48 h (*p* > 0.05 for all comparisons) (Figure 11). Conversely, a statistically significant increase in CI (*p* = 0.04) was found in treated myoblasts after 24 h from procaine administration, while no significant difference was found for the other comparisons at both 24 h and 48 h (*p* > 0.05) (Figure 12).

## 3. Discussion

The scope of this pilot study was to investigate the effects of low concentrations of procaine on the structure and function of mitochondria in two cell types in order to provide information on the cellular events underlying the beneficial outcomes of procaine-based treatments in neural therapy (reviews in [12,13]).

Based on the preliminary tests on cell viability performed on cultured fibroblasts and myoblasts, 0.4 mM (0.01%) procaine was selected for our study as a safe treatment for both cell types. This is consistent with previous findings demonstrating that cultured fibroblasts do not tolerate procaine concentrations higher than 10^−3^ M [61]. In particular, it has been found that 1 to 20 mM procaine causes a calcium overload in mitochondria, inducing mitochondrial dysfunction and a decline in the membrane potential [55]. At these concentrations, procaine acts as an uncoupling agent, reducing the phosphorylation capacity and ATP production and causing the collapse of the mitochondrial membrane potential [56]. Procaine exerts its uncoupling effects by altering lipid packing due to its hydrophobic properties, thus compromising the physical integrity of the inner mitochondrial membrane [56]. Moreover, procaine induces the formation of lipophilic ion pairs with hydrophobic ambient anions; these complexes cycle across the inner mitochondrial membrane, thus causing a proton influx that bypasses ATP synthase and dissipates the proton motive force [62].

On the other hand, at low procaine concentrations (10^−5^ to 10^−4^ M) the uncoupling effect is negligible or even absent [56]. Accordingly, the absence of alterations in the mitochondrial membrane potential observed in fibroblasts and myoblasts treated with 0.4 mM procaine further confirms the safety of the concentration used in the present study.

When the ultrastructural morphology of mitochondria was investigated, no alteration was found in fibroblasts or myoblasts treated with 0.4 mM procaine. This is in agreement with previous observations at TEM on isolated rat liver mitochondria, which demonstrated that low concentrations of procaine (around 10^−5^ M) did not affect the mitochondrial ultrastructural features, whereas concentrations higher than 10^−3^ M produced structural alterations until the organelle disruption [56].

In addition, in the present study we demonstrated by ultrastructural morphometric analysis that, even in the absence of evident morphological alterations, mitochondria may undergo fine modifications related to functional changes. In particular, in fibroblasts treated with 0.4 mM procaine, mitochondria showed a reduction in cristae length at 24 h. Cristae extension is a dynamic feature undergoing continuous changes to preserve mitochondrial structure and functionality in relation to the cell physiological state [63,64]. Cristae length may vary, e.g., to adapt to osmotic changes or to energy metabolism [63]. Since cristae extension has been found to be strictly related to the maximum O_2_ uptake [65], their decrease would suggest a lower functional efficiency in fibroblast mitochondria at 24 h, although this was a transitory event since control values were restored at 48 h. This hypothesis is consistent with the decrease in fibroblast ATP content at 24 h, and by its restoration at 48 h. In addition, it is known that a transient reduction in mitochondrial function may influence cell cycle progression, delaying the S-phase entry without increasing cells death [66,67]; this is consistent with the reduction in BrdU-positive fibroblasts observed in the present study. However, since a direct measurement of respiratory activity was not performed, functional analyses are needed to support this hypothesis.

Conversely, in myoblasts treated with 0.4 mM procaine, mitochondria showed a decrease in the aspect ratio at 24 h (i.e., the organelles became more roundish), and again this modification disappeared at 48 h. It has been reported that slight mitochondrial swelling occurs under physiological conditions to withstand minor stress [68], but also that mitochondria with globular shape generate more ATP than the elongated ones [69]. Both events may be consistent with the increase in cellular ATP observed in myoblasts at 24 h and, although at a lower level, at 48 h. In fact, a stressing condition could reduce cellular ATP consumption, thus causing transient ATP accumulation. It is worth noting that previous biochemical studies showed that procaine has a dual effect on mitochondria, with high concentrations (>10^−3^ M) inhibiting their activity with loss of membrane potential, whereas low concentrations (<10^−3^ M) slightly stimulating the respiratory function [56,57,58,61]; therefore, it could also be hypothesized that myoblast mitochondria increased their activity after 24 h from procaine administration. This hypothesis would also be consistent with the high percentage of BrdU-positive myoblasts observed at 24 h after procaine administration because it is known that ATP stimulates proliferation, accelerating the G_1_ to S phase transition [70]. However, in the absence of measurement of the respiratory activity, the functional interpretation of the above findings remains open.

In order to further evaluate the effect of 0.4 mM procaine on mitochondria in fibroblasts and myoblasts, we quantitatively evaluated the OXPHOS complex subunits that are located along the mitochondrial cristae by Western blot [71]. These enzymes are responsible for the transfer of electrons from a donor to a final acceptor through a series of redox reactions, as well as for the accumulation of protons through CI, CIII, and CIV into the intermembrane space. This energy, stored between the two mitochondrial membranes, is finally used by CV to produce ATP. No modification in the amount of OXPHOS subunits were found in fibroblasts at both 24 h and 48 h, suggesting that the transient reduction in cristae length observed at 24 h is only a functional modulation without a quantitative effect on OXPHOS subunits. On the other hand, in myoblast mitochondria a significant increase in CI and an increasing trend of all other complexes have been found at 24 h. These data suggest that some molecular modifications occur in the mitochondria of myoblasts treated with procaine, although this modification is temporary since no mitochondrial change has been found after 48 h.

In sum, the results obtained in this study show that procaine at a low concentration, although non cytotoxic, influences some mitochondrial features suggestive of functional modifications. However, as stated above, no direct measure of the respiratory activity was performed. In fact, the total ATP level we measured represents not only the ATP produced by respiration but also the glycolytic and total ATP turnover. Therefore, no definite relationship between mitochondrial features and ATP production may be inferred, but the present findings open the way to bioenergetic analyses aimed at clarifying the possible effect of low procaine concentration on mitochondrial respiration.

Interestingly, the observed effects are different in human fibroblasts and mouse myoblasts. These discrepancies could be ascribed to the different species origin of the cell lines; in fact, significant differences in the tolerance to drugs, including procaine, have been reported among animal species [72, 73]. It has also been demonstrated that different cell types may be differently sensitive to procaine [59]. It has been proposed that cell-specific structural features (e.g., the relative abundance of endoplasmic reticulum) could play a key role in the response to procaine [74,75]. In fact, procaine was found to inhibit Ca^2+^ efflux from the endoplasmic reticulum stores, causing anomalous Ca^2+^ accumulation in this organelle and consequent serious cell damage [53,54]. Based on these reports, it may be hypothesized that fibroblasts, equipped with a well-developed endoplasmic reticulum, may undergo more alterations than myoblasts, which are relatively poor in endoplasmic reticulum. Future experiments on Ca^2+^ imaging in different cell types could shed light on the potential role of the endoplasmic reticulum in the response to low procaine concentrations.

## 4. Materials and Methods

### 4.1. Cell Culture and Treatment

Mouse muscle myoblasts C2C12 (ATCC-CRL-1772) were grown in Dulbecco’s modified Eagle’s medium (DMEM), supplemented with 10% heat-inactivated fetal bovine serum (FBS), 2 mM L-glutamine, 1% penicillin/streptomycin, 0.5% amphotericin-B (all reagents were purchased from Gibco, Waltham, MA, USA) at 37 °C in a 5% CO_2_ humidified atmosphere in T75 flasks. Human lung fibroblasts (WI-26, ATCC) were grown in DMEM supplemented with 10% heat-inactivated FBS, 2 mM L-glutamine, 1% penicillin/streptomycin (all reagents were purchased from Gibco) at 37 °C in a 5% CO_2_ humidified atmosphere. Before starting experimentation, both cell lines were stained for DNA with 0.1 µg/mL Hoechst 33342 (Abcam, Cambridge, UK) to exclude mycoplasma contamination.

To treat cells, a 50% solution of procaine hydrochloride in HCl (Farmacia San Carlo, Terre del Reno, FE, Italy) was used. Procaine was diluted in the culture media at the concentrations of 4 mM (0.1%), 2 mM (0.05%) and 0.4 mM (0.01%) to select the maximum safe concentration for both cell lines. To verify that these procaine concentrations did not alter the pH of the culture medium, the pH was measured in three distinct solutions for each concentration. The values ranged from 7.35 to 7.47 at 37 °C in all solutions, demonstrating no pH alteration due to procaine addition.

Therefore, the cells were treated with 4 mM, 2 mM or 0.4 mM procaine when at 70% confluence, and incubated for 1 h at 37 °C in a 5% CO_2_ atmosphere, to mimic procaine clearance physiologically occurring in living organisms through esterase lytic action [76]; this experimental approach has also been used in previous studies [55]. After incubation, the treatment medium was replaced with fresh medium. The effects were evaluated after 24 and 48 h from procaine administration. No differentiation or fusion was observed in C2C12 myoblasts during the experimentation.

### 4.2. Cell Viability Assays

For the MTT assay, the cells were seeded in flat-bottom 96-multiwell plates at a density of 5 × 10^3^ cells/well. Five wells for each condition were seeded, and the assay was performed on samples derived from three independent experiments. The MTT assay was performed 24 h and 48 h after treatment. Briefly, the medium was replaced with 100 μL of 0.5 mg/mL MTT (Sigma, Milan, Italy) in culture medium and incubated for 4 h at 37 °C in an incubator. Then, the MTT solution was removed, formazan crystals were dissolved in 100 μL of dimethyl sulfoxide, and the absorbance was measured at 570 nm with a spectrophotometer microplate reader (ChroMate; RayBiotech, Peachtree Corners, GA, USA). The results were reported as percentages with respect to the control value (set as 100%).

For the trypan blue assay, 6 × 10^4^ cells/well were seeded in flat-bottom 6-multiwell plates. The fraction of dead cells was estimated at 24 h and 48 h after treatment with 0.4 mM procaine by staining for 2 min with 0.1% trypan blue in the culture medium. The assay was performed on samples derived from three independent experiments. Using a Leica DM IL inverted microscope (Leica Microsystems Srl, Buccinasco, MI, Italy) equipped with 20× objective lens, the cells were counted in 10 randomly chosen microscope fields and the percentage of dead (trypan blue-positive) cells was estimated.

### 4.3. S-Phase Evaluation

The percentage of S-phase cells, as a measure of cell proliferation, was evaluated 24 h and 48 h after procaine treatment. Cells (6 × 10^4^ cells per 24 × 24 mm slides) were pulse-labeled with 20 µM BrdU (Sigma) at 37 °C for 30 min and then fixed with 70% ethanol. To partially denature DNA, cells were incubated with 2 N HCl for 20 min at room temperature, then neutralized for 3 min with 0.1 M sodium tetraborate (pH 8.2) (Sigma), washed with phosphate-buffered saline (PBS) and permeabilized for 15 min with PBS containing 0.1% bovine serum albumin and 0.05% Tween-20 (Sigma). The cells were then incubated for 1 h at room temperature with a mouse monoclonal antibody directed against BrdU (BD Diagnostics, Franklin Lakes, NJ, USA) diluted 1:20 in PBS and washed in PBS for 10 min; the cells where then incubated with an Alexa Fluor 488-conjugated anti-mouse secondary antibody (Molecular Probes, Invitrogen, Milan, Italy) diluted 1:200 for 1 h, washed with PBS twice, and stained for DNA with 0.1 µg/mL Hoechst 33342 (Abcam) in PBS for 10 min. Samples were finally mounted with PBS/glycerol 1:1 solution. The percentage of BrdU-positive cell was assessed in 30 randomly selected fields (40×) for each experimental condition. Observation of cell samples was performed using an Olympus BX51 microscope (Olympus Italia S.r.l., Segrate, MI, Italy) equipped with a 100 W mercury lamp under the following conditions: 450–480 nm excitation filter (excf), 500 nm dichroic mirror (dm), and 515 nm barrier filter (bf) for Alexa Fluor 488; 330–385 nm excf, 400 nm dm, and 420 nm bf, for Hoechst 33342. Images were acquired with QICAM Fast 1394 Digital Camera (QImaging, Surrey, BC, Canada) and processed with Image-Pro Plus software (ver. 7.04) (Media Cybernetics, Inc., Rockville, MD, USA). Manual cell counting was performed in samples from three independent experiments by two independent operators. The results were reported as fold change with respect to the control value (set as 1).

### 4.4. Mitochondrial Membrane Potential Assay

The mitochondrial membrane potential (ΔΨm) of the cells was assessed using the lipophilic cation JC-1 (Thermo Fisher, Waltham, MA, USA). At 24 h and 48 h from treatment, the cells (6 × 10^4^ cells per slide) were stained in the dark for 15 min with JC-1 (final concentration in the medium, 2 µM) in a sterile incubator at 37 °C with 5% CO_2_. After double brief washing with PBS, the slides were mounted with PBS/glycerol 1:1 solution. No mitochondrial depolarizing agent was used. Observations and image recording were made with an Olympus BX51 microscope (Olympus Italia S.r.l.) equipped with a 100 W mercury lamp, with the following conditions: 400–500 nm excf, emission (≥520 nm). Images were recorded with an Olympus Camedia C-5050 digital camera (Olympus Italia S.r.l.). JC-1 fluorescence was assessed in 30 randomly selected fields (20×) for each experimental condition. To quantify red and green fluorescence positivity, the software ImageJ (ver. 1.54g, Java 1.8.0; NIH, Bethesda, MD, USA) was used, and the ratio between green and red fluorescence signals were evaluated. Data were obtained from three independent experiments.

### 4.5. Transmission Electron Microscopy Analysis

Morphological and morphometric analyses were carried out using TEM to investigate the effects of procaine treatment on mitochondrial features. A total of 2 × 10^4^ cells were seeded on slides of 22 × 22 mm (five slides per sample) and treated as described above. After 24 h, the cells were fixed for 1 h at 4 °C with 2.5% glutaraldehyde and 2% paraformaldehyde in 0.1 M phosphate buffer, pH 7.4; washed; and post-fixed at 4 °C for 30 min with 1% OsO_4_. Cells were then dehydrated with acetone and embedded in Epon resin as a monolayer [77] (all reagents were purchased from Electron Microscopy Sciences, Hatfield, PA, USA). Ultrathin sections were collected on copper grids and stained with Reynolds’ lead citrate (Electron Microscopy Sciences) for 2 min. Observation of the samples was performed with a Philips Morgagni transmission electron microscope (FEI Company Italia S.r.l., Milan, Italy) operating at 80 kV, and image acquisition was performed with a Megaview III camera (FEI Company Italia S.r.l.). Morphometric analysis of the mitochondrial area, aspect ratio (i.e., the ratio of major mitochondrial axis and minor axis lengths that allows to estimate mitochondrial shape), and cristae extension (i.e., the ratio between lengths of the inner and outer mitochondrial membranes that allows for the estimation of cristae extension independently of the mitochondrial size) was carried out on 20 randomly chosen mitochondria (36,000×) per experimental condition. Data were obtained from three independent experiments.

### 4.6. Cellular ATP Assessment

To detect the cellular ATP levels, a ViaLight^TM^ plus kit (Lonza, Basel, Switzerland) was used. At 24 h and 48 h from treatment, the cells from three independent experiments were harvested by trypsinization, and 1 × 10^4^ cells in 100 μL of medium were transferred to a white 96-well plate. Cellular ATP was extracted by adding 50 μL of cell lysis reagent for at least 10 min, and the bioluminescent signal was generated by adding 100 μL of AMR plus and incubating the plate for 2 min at room temperature. Bioluminescence signal was recorded with a VictorX Multilabel plate reader (Perkin Elmer, Waltham, MA, USA).

### 4.7. Protein Extraction and Immunoblotting of Mitochondrial OXPHOS Complex Subunits

The cell samples were collected 24 h and 48 h post-treatment, immediately frozen in liquid nitrogen and then placed at −80 °C. Proteins were extracted in lysis buffer, i.e., 1 mM Na_3_VO_4_, 1 mM NaF, 2 mM ethylenediaminetetraacetic acid (EDTA), 0.2 mM phenylmethylsulfonyl fluoride (PMSF), 150 mM NaCl, 100× complete protease inhibitor cocktail, and radioimmunoprecipitation assay (RIPA) buffer pH 8.0 (150 mM NaCl, 50 mM Tris-HCl, 1% Igepal, 0.5% sodium deoxycholate, and 0.1% sodium dodecyl sulfate (SDS)). Protein samples (15 μg) were resolved on Tris-glycine 15% SDS–polyacrylamide gel electrophoresis (PAGE) (Bio-Rad, Segrate, MI, Italy), blotted on polyvinylidene fluoride membrane (Life Technologies, Thermo Fisher), and incubated overnight with the following primary antibodies: OxPhos Human WB antibody cocktail (1:500, Invitrogen 45-8199, Thermo Fischer), OxPhos Rodent WB antibody cocktail (1:500, Invitrogen 45-8099, Thermo Fischer) and glyceraldehyde 3-phosphate dehydrogenase (GAPDH) (1:3000, Thermo Fisher Scientific, Milan, Italy). In detail, the OxPhos Human WB antibody is a premixed cocktail of five monoclonal antibodies against Complex I subunit [NDUFB8 (20E9DH10C12)], Complex II subunit 30kDa [SDHB (21A11AE7)], Complex III subunit Core 2 [UQCRC2 (13G12AF12BB11)], Complex IV subunit II [MTCO2 (12C4F12)], and ATP synthase subunit alpha [ATP5A (15H4C4)]. The OxPhos Rodent WB antibody is a premixed cocktail of multiple antibodies against Complex I subunit [NDUFB8 (20E9DH10C12)], Complex II subunit 30kDa [SDHB (21A11AE7)], Complex III subunit Core 2 [UQCRC2 (13G12AF12BB11)], Complex IV subunit I [MTCO1 (1D6E1A8)], and ATP synthase subunit alpha [ATP5A (15H4C4)]. After incubation with the primary antibodies, blots were incubated for 1 h at room temperature with the appropriate horseradish peroxidase (HRP)-conjugated secondary antibody: goat anti-mouse IgG (1:2000, Invitrogen, Fisher Scientific, Milano Italy) or goat anti-rabbit IgG (:2000, Invitrogen, Fisher Scientific, Milano Italy), and developed with a chemiluminescent reagent (ECL, WBLUF0100, Luminata Forte Western HRP substrate, Merck KGaA, Darmstadt, Germany). The signal was detected with the G:BOX F3 GeneSys system version 1.6.9.0 (Syngene, Bangalore, India). Densitometric analysis was performed using ImageJ software (ver. 1.56f, Java 1.8.0; NIH, Bethesda, MD, USA), normalizing the bands to GAPDH levels as a control of equal loading of samples in the total protein extracts. The tests were performed in three independent experiments, a standard procedure in molecular biology. Untreated cells were used as a control.

### 4.8. Statistical Analysis

Data for each variable were pooled according to the experimental condition and presented as mean ± SE). For statistical analysis, the biological replicate was considered the experimental unit. The Shapiro–Wilk test was used to verify the normal distribution of data. Since data were significantly non-normal (*p* < 0.001), statistical differences were determined using the non-parametric Kruskal–Wallis test, and, in case of significance, post hoc group–group comparison was performed with the Mann–Whitney test with Bonferroni correction. The significance was set at *p* ≤ 0.05.

## 5. Conclusions

The present pilot in vitro study provides original evidence that procaine at a low concentration exerts a modulatory effect on some structural and molecular features of mitochondria, reversibly affecting these organelles without causing any damage. It may be therefore hypothesized that, in patients treated with low concentrations of procaine, mitochondrial response could contribute to the reaction of cells—and ultimately of the organism—to diseased conditions, thus accounting for the multiple clinical effects reported in the literature [12,13,19]. To understand the influence exerted by low procaine concentrations on mitochondrial respiratory activity, functional investigations on a large sample size (to provide high statistical power for detecting even modest molecular modifications) are planned.

In addition, our study suggests that different cell types or those from different species diversely respond to the same procaine concentration. This aspect particularly deserves further investigation because it may affect the therapeutic outcomes.

The in vitro cellular model used in our study proved to be suitable for mechanistically analyzing cellular events following procaine treatment, although it is far from the complexity of the whole organism. The present findings should therefore be considered as basic knowledge for additional studies on ex vivo and in vitro models aimed at understanding the mitochondrial response to low procaine concentrations under conditions closer to a living organism. This will advance scientific knowledge on the biological pathways responsible for the beneficial effects of procaine-based therapies, which are matter of lively debate in the medical community. This knowledge will also provide clinicians with a robust scientific basis for improving protocols for effective and personalized treatments.

## Data Availability

The original contributions presented in this study are included in the article. Further inquiries can be directed to the corresponding author.
